# Monitoring the Multiple Stages of Climate Tipping Systems from Space: Do the GCOS Essential Climate Variables Meet the Needs?

**DOI:** 10.1007/s10712-024-09866-4

**Published:** 2025-02-18

**Authors:** S. Loriani, A. Bartsch, E. Calamita, J. F. Donges, S. Hebden, M. Hirota, A. Landolfi, T. Nagler, B. Sakschewski, A. Staal, J. Verbesselt, R. Winkelmann, R. Wood, N. Wunderling

**Affiliations:** 1 Earth Resilience Science Unit and Earth System Analysis, Potsdam Institute for Climate Impact Research, Member of the Leibniz Association, Telegrafenberg 31A, 14473 Potsdam, Germany; 2 b.geos, Industriestrasse 1A, 2100 Korneuburg, Austria; 3 Swiss Federal Institute of Aquatic Science and Technology, 8600 Dubendorf, Switzerland; 4 Stockholm Resilience Centre, Stockholm University, Albanovägen 28, 106 91 Stockholm, Sweden; 5 High Meadows Environmental Institute, Princeton University, Princeton, NJ 08544 USA; 6 Future Earth Secretariat, 11418 Stockholm, Sweden; 7 Group IpES, Department of Physics, Federal University of Santa Catarina, Florianópolis, 88034-102 Brazil; 8 Department of Plant Biology, University of Campinas, Campinas, 13083-970 Brazil; 9 National Research Council of Italy, CNR-ISMAR-Roma, 00133 Rome, Italy; 10 ENVEO Environmental Earth Observation Information Technology GmbH, Fürstenweg 176, 6020 Innsbruck, Austria; 11 Copernicus Institute of Sustainable Development, Utrecht University, Princetonlaan 8a, 3584 CB Utrecht, the Netherlands; 12 Belgian Science Policy Office (BELSPO), Simon Bolivarlaan 30 Bus 7 Boulevard Simon Bolivar 30 Bte 7, 1000 Brussels, Belgium; 13 Laboratory of Geo-Information Science and Remote Sensing, Wageningen University and Research, Droevendaalsesteeg 4, 6708 PB Wageningen, The Netherlands; 14 Institute for Physics and Astronomy, University of Potsdam, Potsdam, 14476 Germany; 15 Integrative Earth System Science, Max Planck Institute of Geoanthropology, Jena, 07745 Germany; 16 Met Office Hadley Centre, FitzRoy Road, Exeter, EX1 3PB UK; 17 ECSAT, European Space Agency, Harwell, Didcot OX11 0FD UK; 18 Center for Critical Computational Studies, Goethe University, Theodor-W.-Adorno-Platz 1, Frankfurt am Main, 60629 Germany

**Keywords:** Tipping points, Earth Observation, GCOS, Essential Climate Variables

## Abstract

Many components of the Earth system feature self-reinforcing feedback processes that can potentially scale up a small initial change to a fundamental state change of the underlying system in a sometimes abrupt or irreversible manner beyond a critical threshold. Such tipping points can be found across a wide range of spatial and temporal scales and are expressed in very different observable variables. For example, early-warning signals of approaching critical transitions may manifest in localised spatial pattern formation of vegetation within years as observed for the Amazon rainforest. In contrast, the susceptibility of ice sheets to tipping dynamics can unfold at basin to sub-continental scales, over centuries to even millennia. Accordingly, to improve the understanding of the underlying processes, to capture present-day system states and to monitor early-warning signals, tipping point science relies on diverse data products. To that end, Earth observation has proven indispensable as it provides a broad range of data products with varying spatio-temporal scales and resolutions. Here we review the observable characteristics of selected potential climate tipping systems associated with the multiple stages of a tipping process: This includes i) gaining system and process understanding, ii) detecting early-warning signals for resilience loss when approaching potential tipping points and iii) monitoring progressing tipping dynamics across scales in space and time. By assessing how well the observational requirements are met by the Essential Climate Variables (ECVs) defined by the Global Climate Observing System (GCOS), we identify gaps in the portfolio and what is needed to better characterise potential candidate tipping elements. Gaps have been identified for the Amazon forest system (vegetation water content), permafrost (ground subsidence), Atlantic Meridional Overturning Circulation, AMOC (section mass, heat and fresh water transports and freshwater input from ice sheet edges) and ice sheets (e.g. surface melt). For many of the ECVs, issues in specifications have been identified. Of main concern are spatial resolution and missing variables, calling for an update of the ECVS or a separate, dedicated catalogue of tipping variables.

**Article Highlights**

- The diversity of potential climate tipping systems brings about a variety of variables, spatial and temporal scales required to be observed
- Many complementary remote sensing products can monitor the different stages that tipping systems pass through
- The GCOS ECVs capture many tipping-relevant variables and scales, but are often not sufficiently specified and some are missing, calling for a partial update of the GCOS ECVs or a new set of Essential Climate Tipping Variables

## Introduction

Climate tipping elements are large-scale subcomponents of the Earth system with the potential to undergo nonlinear and often irreversible state shifts under increasing anthropogenic pressures (Armstrong McKay et al. 2022; Lenton et al., 2023; Rockström, et al., 2023; Wang et al., 2023). They are associated with large risks for human societies and the Earth system overall, and are hence decisive for human development within safe and just Earth system and planetary boundaries (Rockström, et al., 2023; Richardson et al., 2023). Among potential climate tipping elements are biosphere components such as the Amazon forest system (Flores et al., 2024), circulatory patterns such as the Atlantic Meridional Overturning Circulation (AMOC, an ocean current system; Rahmstorf 2024), and cryosphere systems such as the ice sheets on Greenland and Antarctica (Gregory et al., 2020; Fox-Kemper et al. 2021). For all these systems, self-reinforcing feedback dynamics could not only amplify anthropogenic change but even drive further change in a self-sustained manner. Thereby, compared to gradual changes in the Earth system, tipping points pose significant additional risks by leading to nonlinear and sometimes abrupt and irreversible change. For example, certain feedback processes in ice sheets amplify human-caused destabilisation and could grow to an extent that irreversibly continues to drive further ice sheet disaggregation, potentially leading to large-scale sea level rise on the order of several metres possible within 2 °C of global warming (Pattyn and Morlighem 2020). Several of the climate tipping elements have been projected to be at increasing risk of destabilisation if global warming levels overshoot 1.5 °C (Bustamante et al 2023; Bauer et al., 2023; Wunderling et al. 2023a, b; Armstrong McKay et al. 2022). Moreover, there are further smaller-scale tipping or regime shift processes such as lakes tipping from a clear to a turbid water state (Scheffer et al. 2001), or dramatic decreases of ocean oxygen concentrations (Rocha et al. 2018) that are important for their feedbacks on the Earth system and as natural resources. As they obey similar system dynamics as the large-scale climate tipping elements, we here refer to both categories as *tipping systems*.

While substantial community efforts on systematic Earth system modelling and analysis of climate tipping systems and their impacts are underway (e.g. through the Tipping Points Modelling Intercomparison Project, TIPMIP), observational efforts that leverage global-scale remote sensing data are playing an increasingly important role (Lenton et al. 2024). Satellite-based data products have been used to characterise tipping systems at risk, for example by detecting the accelerating loss of Antarctic Peninsula ice shelves and ice sheets, helping to identify the destabilisation of the West Antarctic ice sheet (Mouginot et al 2014), and have been used for monitoring biosphere tipping systems’ resilience over space and time (Verbesselt et al 2016; Boulton et al 2022; Dakos et al. 2024). Lenton et al. (2024) call for a smarter and combined use of remote sensing data in a tipping point sensing system, to detect and forewarn of tipping risk and support policymaking and environmental management across scales. This requires, among other things, an improvement in the analysis-ready data available for Earth system monitoring and reporting of the changes. There are existing efforts to harmonise the characterisation of Earth’s climate via so-called Essential Climate Variables (ECVs), which are physical, chemical or biological variables (or a group of linked variables) established by the Global Climate Observing System (GCOS) (WMO, 2022a). They are quantified by measurable parameters called ECV products. ECVs are selected within the GCOS programme of the World Meteorological Organization (WMO) which collects and documents the data needs for monitoring the climate system and assessing the impacts of climate variability and change (WMO 2022b). ECVs have been also previously reviewed for specific environments, e.g. mountain regions (Thornton et al. 2021) for which more than 20 variables have been found not considered in GCOS.In this contribution, we compare the currently observed ECVs with the requirements to more stringently monitor Earth systems with potential tipping points.

### The Stages of a Tipping System

Tipping systems are characterised by positive feedbacks, which become self-propelling beyond a critical threshold of forcing (tipping point). This means that, beyond the tipping point, the dynamics of a tipping system are dominated by self-amplifying feedbacks, which drive the state of the system from one stable state to another. More precisely, at the tipping point a small change in a critical system-dependent forcing variable (e.g. temperature, precipitation or their combination) is sufficient to push the tipping system across its critical threshold leading to significant state change in the affected system (Armstrong McKay et al. 2022; Lenton et al. 2008; Scheffer, 2001).

**Fig. 1:**
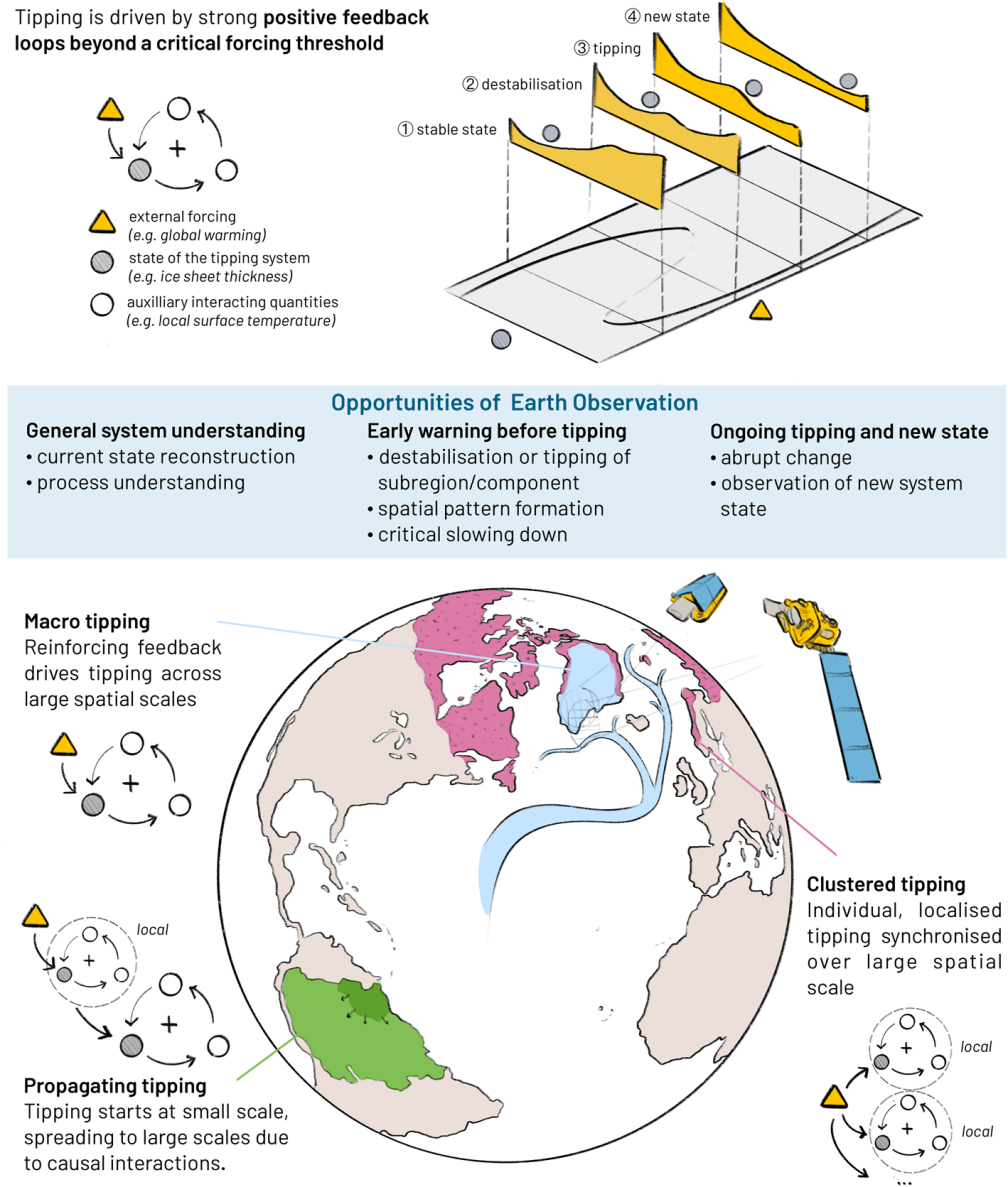
Earth observation opportunities to monitor tipping across scales. Tipping occurs in systems where positive feedback loops maintain change beyond a critical forcing threshold. These dynamics can play out on different spatial scales depending on the range of the feedbacks – tipping on large scales can be the consequence of either a feedback spanning a wide (macro) range, a regional feedback that propagates to larger scales, or local feedbacks that act independently but are subject to the same external conditions and thereby synchronise over large spatial extents (see Lenton et al. 2024)

We can broadly divide the journey of a tipping system throughout its state change into four stages of tipping, giving rise to various Earth observation opportunities along these stages (see Fig. 1, top). As an illustrative and simplified example of these four stages, a tipping system (grey marble in Fig. 1) can be imagined to reside in a potential landscape (ball-and-cup diagram) with two minima (Scheffer and Stephen 2003).

1. In the first stage, the tipping system is located in its *stable state* and is relatively far away from its tipping point.
2. In the second stage, the tipping point is approached through increased forcing (yellow triangle in Fig. 1), *destabilising* the currently assumed stable state. This stage can under certain conditions be characterised by measurable temporal and spatial early-warning signals (EWS) as resilience decreases and return times to the original stable equilibrium following stochastic shocks increase (Scheffer et al. 2009). In the illustrative potential landscape, this is because the potential valley where the tipping system still resides becomes shallower as the forcing increases.
3. The third stage describes a tipping system that is in the *ongoing process of transitioning* from one stable state to another. In this stage, the formerly stable potential minimum becomes unstable and the grey marble rolls over to a tipped / alternative state.
4. In the final stage four, the tipping system is characterised by the *completed tipping process* and resides in the new state.

While we focus on so-called bifurcation-induced tipping driven by external forcing or internal slow change of a critical variable or parameter (slow in the sense of time-scale separation), other types of tipping are conceivable. For example, for noise- and rate-induced tipping (Ashwin et al. 2012) the state change is triggered by stochastic variability or rapid external forcing. While this may have implications for observability of early-warning signals (Swingedouw et al 2020; Dakos et al. 2024), the underlying positive feedbacks leading to tipping are analogous.

In earlier literature, large tipping systems have often been viewed as simple bistable systems (e.g. the Greenland Ice Sheet as a system fully intact vs collapsed, or the Amazon forest system as intact forest vs savanna state) governed by a single equation (Scheffer et al. 2009; Brummitt et al. 2015; Wunderling et al. 2021). However, reality is more intricate and multiple stable states may exist, not least due to the development of spatial patterns and regionally different properties (Robinson et al. 2012; Rietkerk et al. 2021; Lenton et al., 2023). The mechanisms underlying tipping systems can be broadly divided into three different types because of their varying feedback structure (Lenton et al. 2024) as shown in Fig. 1.

- *Macro tipping:* The system has a feedback that plays out on a system-wide scale and leads to large-scale tipping. Examples of macro tipping are the large-scale instability of the ice sheets on Greenland and West Antarctica due to the melt-elevation feedback (see Sect. 2.1).
- *Propagating tipping:* The system has coupled components, such that destabilisation of some parts propagates to the larger scale in a cascading manner. This means local failures can spread system-wide. An example for propagating tipping is the Amazon rainforest, where localised dieback in the northeast may propagate southwest due to a reduction in moisture recycling feedback (see Sect. 2.3).
- *Clustered tipping:* Localised, individual and disconnected tipping events are triggered over large spatial extents by an external forcing. If the external forcing is synchronised over large spatial extents, large parts of a tipping system disintegrate at the same time even without any large feedback that connects these disjoint parts (contrary to macro tipping). An example for clustered tipping is the abrupt thaw of Arctic permafrost (see Sect. 2.4) or dieback of tropical coral reefs, with highly localised dynamics on the level of individual melt ponds or corals.

Lastly, since each tipping system is embedded in the interacting Earth system, they may influence and further destabilise each other, and interact with non-tipping systems (which can also help stabilise them) (Wunderling/von der Heydt et al., 2024).

In summary, given this diversity, tipping unfolds in a huge variety of spatial and temporal scales in the Earth system. At the same time, the stage and type of the tipping system are decisive for its dynamics. In particular, even within one tipping system, dynamics related to tipping (initial state, early-warning signals, the actual tipping transition and the established new state, see Fig. 1) play out in many different dimensions and variables and on various spatial as well as temporal scales. Therefore, rigorous Earth observation efforts are necessary in order to swiftly measure impending changes within Earth system components relevant for planetary stability, to inform policy making and environmental management, in particular for climate mitigation and adaptation.

### Earth Observation for Climate Change Monitoring

Climate data records (CDR) characterise the evolution of global climate and comprise data from in situ instruments, paleoclimate, airborne and satellite sensors. Crucially, they need to be sufficient in time span, consistency and continuity to detect climate trends—typically several decades are necessary (Yang et al. 2016). The Global Climate Observing System (GCOS) is tasked with defining the requirements for CDRs that are essential for monitoring global climate change, the ‘Essential Climate Variables’ (ECVs). With the advances in satellite observations from passive and active sensors, on both geostationary and polar orbiting missions, satellites make an indispensable contribution to global climate monitoring efforts (Lenton et al. 2024), and support about 60% of the GCOS ECVs (WMO, 2022a). ECV requirements are determined by community consultation and expressed in terms of spatial and temporal resolution, measurement uncertainty, stability and timeliness (a newly introduced aspect which identifies how fast data records should be made available after measurement). Recently (end of 2022), GCOS proposed new ECVs and their requirements (WMO, 2022b), alongside major practical actions to be undertaken in the next 5–10 years within the GCOS Implementation Plan (WMO, 2022a). The European Organisation for the Exploitation of Meteorological Satellites (EUMETSAT) hosts a satellite-derived ECV inventory on behalf of the Committee on Earth observation satellites (CEOS) and Coordination Group for Meteorological Satellites (CGMS), which currently lists more than 800 existing and 300 planned data records (ECVInventory v4.1).

Here we discuss these new GCOS ECVs requirements from the perspective of tipping point science. To this end, we review how remote sensing provides insights on the status and key positive feedback processes of selected potential tipping systems, and discuss how satellite observations may serve to monitor systems losing in resilience and approaching criticality (early warning of tipping). In this work, we build on first steps done on evaluating the remote sensing potential for tipping points monitoring (Lenton et al. 2024) by synthesising observational needs involved with the different tipping stages, and checking the ECV requirements set by GCOS (WMO, 2022b) and the EUMETSAT inventory (ECVInventory v4.1) to identify gaps. We constrain the in-depth analysis on a selection of global core tipping elements (Armstrong McKay et al. 2022), including the ice sheets, the Amazon forest system, ocean circulations and high-latitude permafrost. Finally we complement our analysis with two selected tipping systems (lake stratification and ocean oxygen). They are exemplary for the wide class of additional potential tipping systems beyond the global core tipping elements, which may undergo large and irreversible changes in response to ongoing climate change (Rockstrom et al., 2009, Lenton et al. 2008, Heinze et al. 2021) but are currently regarded as largely uncertain due to limited evidence (Armstrong McKay et al. 2022). Overall, our selection of systems provides study cases out of many different Earth system realms—land-based and marine biosphere, ocean circulations and freshwater systems, and cryospheric systems. This shortlist may be extended in future work.

## Selected Biophysical Tipping systems

### Ice Sheets

#### Tipping System and Feedback Mechanisms

The ice sheets over Greenland and Antarctica cover extensive terrestrial regions, holding the majority of the Earth’s freshwater. On the one hand, these systems exhibit high inertia, such that the bulk of induced change may unfold only in the order of centuries. On the other hand, they are strongly interacting with the atmosphere, oceans and biosphere, and are significantly susceptible to global warming. Continued, accelerating melt and disintegration of the ice sheets is contributing to sea level rise and alteration of regional climate (Fox-Kemper et al. 2021). Based on modelling evidence and reconstructions of paleo data, the ice sheets have been identified as tipping elements in previous assessments (Armstrong McKay et al. 2022; Lenton et al. 2023; Wang et al. 2023).

**Fig. 2:**
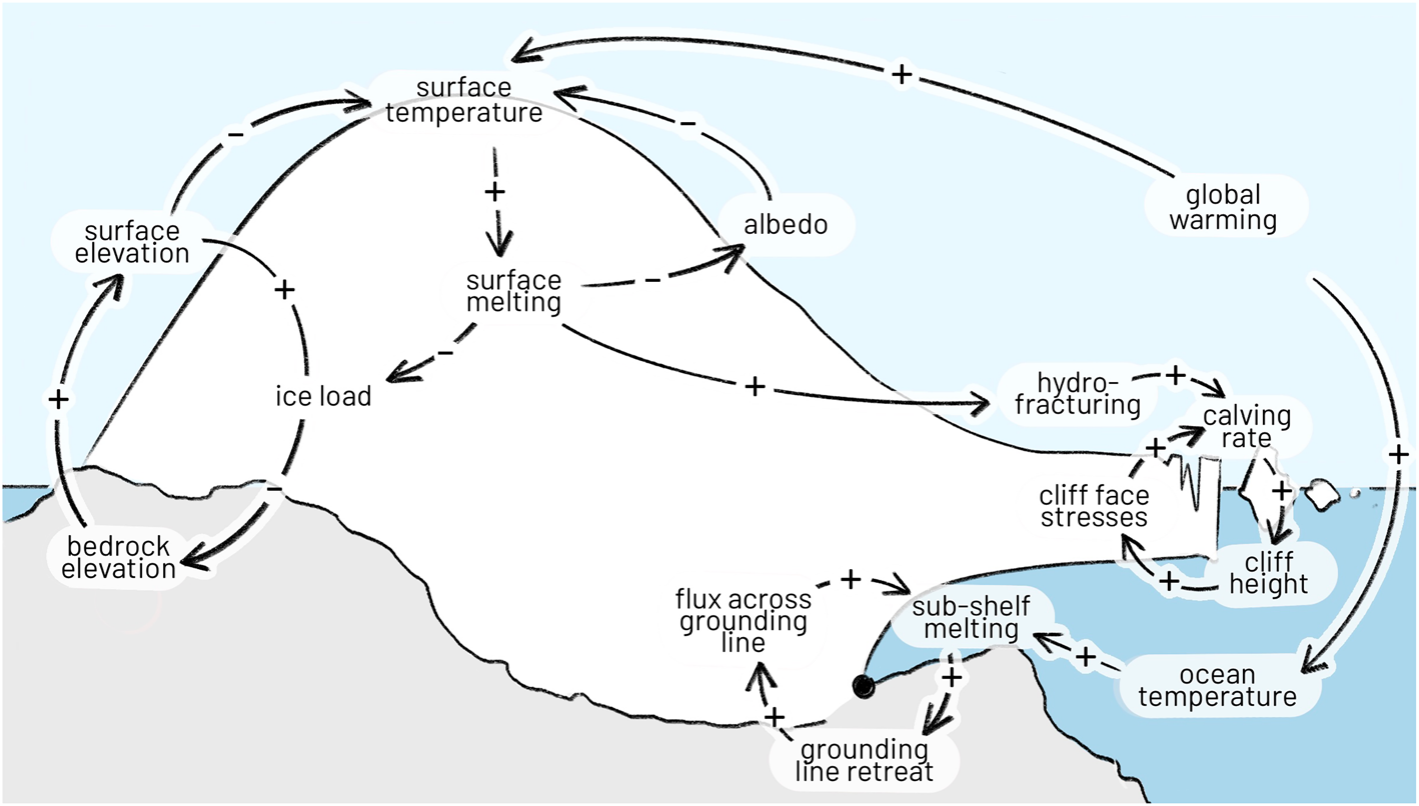
Dominant feedback loops in ice sheets. Based on Zeitz (2022) and Lenton et al. (2023)

A series of feedbacks determine the long-term dynamics of the ice sheets, with positive ones potentially fuelling self-sustained melt beyond critical thresholds. As the ice sheet surface gets to lower altitudes due to melting, it is exposed to higher surface temperatures, which in turn lead to an increased melting and so on. Modelling and evidence from paleo data show that this melt-elevation feedback could lead to self-sustained ice sheet disintegration, and might have driven Greenland ice sheet collapse in the past (Robinson et al. 2012; Levermann and Winkelmann 2016). On time scales of millenia, this is partially compensated by glacial isostatic adjustment, in which the compressed bedrock rises vertically with decreasing ice load, acting as a negative feedback (Whitehouse et al. 2019). Surface melt can be amplified by an albedo feedback, in which the melting ice exposes the underlying ground material with lower albedo, leading to increased solar radiation absorption ensuing higher ambient surface temperature and melt (Box et al. 2012). Concerning marine-terminating glaciers, a potential mechanism accelerating melt is the marine ice sheet instability (MISI): Warming oceans increase sub-shelf melting in the shelf regions, leading to the retreat of the grounding lines (the boundary where an ice sheet transitions from being grounded on bedrock to floating on the ocean). On retrograde grounds (i.e. the bedrock descends land-inwards, see Fig. 2), the consequence is a larger ice sheet cross section at the grounding line, leading to an enhanced outflow. The ensuing further grounding line retreat closes the loop and may self-sustain the sub-shelf melt until pro-grade conditions (Schoof 2007; Feldmann and Levermann 2015; Garbe et al. 2020). A more speculative feedback, however with potentially significant consequences, is marine ice cliff instability (MICI). It is posited that the collapse of marine-terminating ice sheets may lead to high, unstable cliffs at the edge with increased iceberg calving rate, which in turn maintains the creation of these unstable cliffs (Bassis and Walker, 2011; DeConto and Pollard 2016). Mechanisms opposing the retreat of ice sheets include buttressing by ice shelves (Gudmundsson et al. 2012) or other states of ice (e.g. mélange of sea ice and icebergs, see Schlemm and Levermann 2021; Robel 2017), in which the three-dimensional geometry of the ice sheet and its surrounding (e.g. narrow fjords) can provide mechanical stability.

Alongside these major feedback loops, there are other processes concerning the feedback of the ice sheet on the ambient atmosphere and ocean, which in turn may affect the melting. For example, changes in ice sheet topography will modify the orographic precipitation around the margins, likely resulting in less precipitation at the margins but in an increase of precipitation towards the interior of the ice sheet. The net effect of this precipitation change is estimated to be negative for the Greenland Ice sheet (Hakuba et al. 2012). The surface mass balance in Antarctica is mostly determined by advection of the surrounding ocean, such that changes in the precipitation over the Southern Ocean have a large impact on the ice sheet dynamics. However, different satellite and reanalysis products diverge on the assessment of that precipitation (Bromwich et al. 2011). Similarly, the freshwater inflow into the ocean increases stratification, with potentially far-reaching consequences. The resulting increase in stratification could, for example, weaken the AMOC, which in turn would lead to cooling over Greenland and a decreased melt, hence potentially constituting a negative feedback (Wunderling/von der Heydt et al., 2023). Similarly, the retreat in Antarctic ice shelf fronts may lead to modulation in regional ocean dynamics and heat transport, presumably acting as a negative feedback (Yoon et al 2022). In summary, there needs to be a close monitoring of not only the ice sheet itself but also key ambient system variables, e.g. sea surface salinity and surface wind vectors. This will improve the understanding of how potential ice sheet tipping may emerge from the interplay of the ice sheet and ambient systems.

#### Earth Observation of Potential Tipping

##### Introduction: Monitoring the Ice Sheets from Space

Satellite-based Earth observation, applying various measurement principles, has been indispensable in the study of ice sheets. Laser altimetry applies a ranging technique to measure the distance between satellite and ice surface with high accuracy. Repeated observations are used to retrieve changes in surface elevation of ice sheets at high spatial resolution (e.g. 100 m nominal resolution using ICESat 1 and 2). Radar altimetry exploits a similar measurement principle but uses microwave pulses, operating at lower resolution, but a key benefit is that microwave frequencies can penetrate clouds and provide all-time monitoring. It is used at different frequencies (Ku-band or S-band) and modes (e.g. on ERS-2, Envisat, CryoSat-2 and Copernicus Sentinel-3). Synthetic aperture radar (SAR) uses a radar antenna to generate high-resolution images of the ice surface by exploiting the Doppler effect of the reflected radar signals to synthesise a large antenna, thus improving the spatial resolution that can be obtained. SAR images can be used to measure ice sheet velocity by tracking features on the ice surface over time (feature tracking) or by comparing the phase of the radar signals from two consecutive images (interferometry) to measure surface displacement. SAR images can also be used to measure the grounding zone by detecting the tidal flexure zone where the ice sheet transitions from grounded to floating (through differential interferometry). Since 1991 several SAR missions including ERS 1/2, ENVISAT ASAR, RADARSAT-1/2, TerraSAR-X and TanDEM-X, ALOS PALSAR and Copernicus Sentinel-1 have been launched, allowing continuous observation of the ice sheets at different radar frequencies (L-band, C-band, or X-band) and modes (single, dual, or quad polarisation) with high spatial resolution and with increasing temporal frequency. For example, since the launch of the Copernicus Sentinel-1 satellites in 2014 and 2015 the margins of Antarctica and Greenland have been continuously observed with weekly to 12 days repeat observations, enabling detection of seasonal and even monthly variations of ice dynamics and calving events. Active (SAR, Scatterometer systems) and passive microwave sensors enable also to estimate surface melt spatial extent based on the significant reduction of backscatter in the presence of liquid water in the snow layer. Scatterometer backscatter maps and passive microwave data enable monitoring of the temporal dynamics of surface melt at daily or even sub-daily time steps but with coarse resolution only (several km to tens of km). SAR complements this information with less frequent (weekly to biweekly) but spatially detailed information suitable for outlet glaciers in complex mountainous regions (e.g. Antarctic Peninsula). The trade-offs between these approaches are summarised by de Roda Husman et al. (2024) who merge the multiple satellite records (MetOp ASCAT C-band (5.255 GHz) radar and 19 GHz passive microwave records from SSMIS) using machine learning to produce a prototype, Antarctic-wide surface melt extent record.

A very different method for monitoring ice sheets is gravity recovery which involves the motion of satellites influenced by the present (ice) masses. GRACE and GRACE-FO use a K-band microwave ranging system and a GPS receiver to measure the inter-satellite distance and position which allows retrieval of mass changes on Earth after correcting for non-gravitational drag effects (measured on-board by accelerometers). The upcoming ESA-NASA MAGIC mission aims to monitor Earth’s gravitational field at a resolution of 100 km every 3 days (Haagmans and Tsaoussi 2020).

In essence, based on these measurement principles, there are three methods to derive the total mass balance of the ice sheets from remote sensing products: (i) gravimetry, (ii) altimetry, (iii) input/output method exploiting measurements of the ice surface velocities (via feature tracking) and surface mass balance estimates (derived from altimetry and ice sheet density models) (IMBIE, 2020). Recent assessments confirmed continued mass loss in both ice sheets. On Greenland, enhanced surface melting and iceberg calving are major drivers of mass loss (King et al. 2020; IMBIE, 2020). Several outlet glaciers have experienced significant retreats and acceleration in the past two decades. For instance, Jakobshavn Isbrae, Helheim Glacier, and Kangerdlugssuaq Glacier have shown a dynamic response to oceanic and atmospheric forcing, as detected by SAR interferometry and feature tracking using data from ERS-1/2, Envisat, and Sentinel-1 satellites (Joughin et al., 2004; King et al. 2020; IMBIE, 2020; Smith et al. 2020). In Antarctica, most melting is observed on the Antarctic Peninsula, and is dominated by ocean-induced sub-shelf melting. The collapse of the Larsen A (1995) and B (2002) ice shelves on the Antarctic Peninsula were captured by ERS-1/2 and RADARSAT-1 SAR images and revealed a rapid disintegration of the ice shelves into thousands of icebergs (Rott et al. 1996; Scambos et al., 2004). In the Amundsen Sea sector of West Antarctica, the Pine Island Glacier and the Thwaites Glacier are also undergoing rapid changes due to enhanced sub-shelf melt (Pritchard et al., 2012). These processes have been monitored by radar altimetry and gravity recovery using data from ERS-1/2, Envisat, CryoSat-2, and GRACE satellites (Rignot et al., 2019; IMBIE, 2020; Scambos et al., 2004) and by laser altimetry with the ICESat satellites. Future missions such as the Copernicus Polar Ice and Snow Topography Altimeter (CRISTAL) mission and the NASA-ISRO SAR (NISAR) mission will provide more accurate and high-resolution measurements of ice sheet elevation and mass changes, which are essential for improving ice sheet models and projections (Markus et al. 2017; Flechtner et al. 2016) (Table 1).

**Table 1:** Parameters relevant to ice sheet tipping, and corresponding GCOS-ECVs

Variable related to tipping dynamics	GCOS-ECV parameters		Comment/gap(s)	Suitable?
	Variables	Specs (resolution)		
**General ice sheet state**				
Ice mass*	Ice volume change	Threshold annual at 50 km, goal monthly	Goal spatial resolution undefined	
Ice velocity and Ice velocity change*	Ice velocity	Threshold annual at 1 km, goal monthly at 50 m	No specifications available for velocity change	
**Particularly for melt-elevation feedback (incl glacial isostatic adjustment)**				
Surface elevation	Surface Elevation Change	Threshold annual at 100 m, goal monthly	Goal spatial resolution undefined	
Surface air temperature	Air temperature (near surface)	Threshold 500 km and 3 h, goal 10 km hourly		
Surface melt extent/ melt duration	–	–	Not included; 10 km, daily (IGOS Cryosphere Theme Report 2007, page 86/87)	M
Vertical land motion	–	–	Not included	M
**Particularly for melt-albedo feedback**				
Surface albedo	Spectral and Broadband (Visible, Near Infrared and Shortwave) DHR and BHR6 with Associated Spectral Bidirectional Reflectance Distribution Function (BRDF) Parameters	Threshold 5 days at 250 m, goal daily at 10 m		
Grounding line (zone) location*	Grounding Line (zone) Location and Thickness	Threshold annual at 1 km, goal 100 m	Goal temporal resolution un defined	
Mass Flux across the grounding line	–	–	Not included	M
Sub-shelf topography	–	–	Not included	M
**Particularly for marine ice cliff instability**				
Calving rate / calving front position*	–	–	Not included	M
Areas of hydrofracturing	–	–	Not included	M
Sea ice properties*	Sea ice thickness	Threshold 50 km and monthly, goal 1 km and daily		
	Sea ice concentration	Threshold 50 km and monthly, goal 1 km and better than daily		

A star (*) indicates what is directly addressed in ESA CCI baseline projects. The last column indicates where the GCOS-ECV parameters do not match the specific needs of tipping science either by insufficient or undefined specs (X) or entirely missing ECV (M)

##### Insights Into key Feedback Loops Relevant for Tipping

For marine-terminating ice sheets, the migration of the grounding line (zone) is a sensitive measure of ice sheet stability and potential onset of the MISI feedback. It can be inferred from observational data (Hogg et al., 2018) by determining the vertical displacement of floating ice at tidal frequencies through InSAR (Rignot et al. 2011; Scheuchl et al. 2016), altimetry (e.g. Brunt et al., 2011), or by analysing shadows in optical satellite imagery (Scambos et al. 2007). A recent study based on InSAR data suggests that the rapid retreat of Pope, Smith and Kohler glaciers in the Amundsen bay observed in the past decades happened on retrograde bed slopes (Milillo et al., 2022). This serves as a strong study case for MISI, and is therefore especially relevant for the neighbouring larger Pine and Thwaites glaciers. These glaciers share similar characteristics in terms of underlying physical processes but hold more than a metre of global sea level rise potential (Fox-Kemper et al. 2021; Milillo et al., 2022).

Inherent to the nature of the feedback, it is challenging to directly observe MICI (Needle and Holschuh, 2023). High temporal sampling and a complementary suite of remote sensing products are needed to monitor the rapid breakup of ice sheet fronts and the variety of factors that determine their geometric (in-) stability. In particular, it is necessary to not only assess the calving front position (see e.g. Greene et al., 2022), calving rate and the ice velocity but also surface properties (such as areas of hydrofracture) along with the regional conditions on ground (bed topography) and sea (presence of sea ice or mélange). Individual study cases such as Crance glacier’s retreat following the collapse of the Larsen B ice shelf can help to constrain model parameterisations of critical ice cliff heights (Needle and Holschuh, 2023).

Variables involved in the feedbacks associated with elevation and albedo are in principle more accessible via remote sensing. The ice sheet surface elevation is a critical parameter for the melt-elevation feedback, and is made readily available from radar altimetry (from ERS1/2, Envisat, and CryoSat-2) within the ESA CCI programme (Sandberg Sørensenet al. 2018; IMBIE, 2020). For mass balance estimates, changes in surface elevation need to be corrected for density and the isostatic adjustment, i.e. requiring independent assessments of vertical ground motion. Finally, surface air temperatures and melt rates require close monitoring to draw conclusions on the onset of this positive feedback. Characterisation of the melt-albedo feedback needs measurements of surface melt extent, (near-) surface air temperature and surface albedo.

As outlined in the Introduction, bistable tipping systems are expected to have increasingly long recovery times from external perturbations when approaching their tipping point. This is expressed as increasing auto-correlation in spatial or temporal data. However, the characteristic time scales for ice sheet-related processes are naturally orders of magnitude longer than for e.g. living systems, such that a complete disintegration would take centuries to millennia and fluctuations play out on annual or decadal time scales. This makes it challenging to observe early-warning signals—in fact, all studies of critical slowing down in ice sheets have been based on either station data (Boers and Rypdal 2021) or modelling exercises (Rosier et al., 2021).

However, remote sensing is invaluable to make sense of the past (and ongoing) collapse and rapid retreat events, leading to better modelling and predictive capacities. Several studies analysed in hindsight the observational evidence leading up to the collapse of the Larsen ice shelves, attributing the rapidness to e.g. hydrofracture networks (Banwell et al. 2013). Similarly, substantial thinning of the glacier terminus before the onset of rapid retreat could be identified in all regions of the Greenland Ice sheet (King et al. 2020). These analyses were underpinned by significant progress in system understanding and modelling capabilities, pointing towards a promising direction for early warning for ice sheet collapse. Indeed, improved assimilation of remote sensing products with modelling could serve as a transient forecast, similar to recent advances in sea ice extent forecasting (Navarro et al. 2022).

##### Discussion: Gaps in GCOS and Recommendations

The different remote sensing methods for the ice sheets each have their respective strengths and drawbacks. To infer ice mass from altimetry, accurate models of snow accumulation and compaction are required to infer firn layer thicknesses. This is informed by surface mass balance models, which in turn require accurate capturing of regional climate, thereby heavily relying on atmospheric and surface ECVs. The input/output method additionally requires estimates of frontal ablation, which is limited by the outlined challenges in estimating hard-to-observe processes such as sub-shelf melting and rapid calving. As a direct measurement of mass, gravimetry is relieved from some of these difficulties, but it is particularly sensitive to glacial isostatic adjustment (GIA). To disentangle this signal from changes in the ice sheet mass, accurate modelling of GIA is needed, and/or assimilation of complementary approaches (e.g. GPS, altimetry) with a different GIA sensitivity (van der Wal et al. 2015). Closely related are the condition and dynamics of regional topography, as it has significant implications for MISI (e.g. through retrograde slopes or cavities) and ice sheet geometry potentially determining MICI. The vertical land motion as well as sub-shelf topography are therefore key observables which are currently missing from the GCOS-ECV portfolio.

Recent intercomparisons (King et al. 2020; IMBIE, 2018, 2020) show fairly consistent results between the different total mass balance estimation approaches in many regions. However, large uncertainties emerge from the poor sampling (both limited in situ data and spatial coverage) of some regions such as East Antarctica (Rignot et al. 2019) or the grounding zones of fast flowing glaciers. At the general level, it seems that such issues are increasingly recognised, such that the GCOS 2022 Implementation Plan, identifies “improved ECV satellite observations in polar regions” as an emergent need (WMO, 2022a). However, some specific associated variables remain excluded or poorly defined in the GCOS-ECV portfolio (WMO, 2022b). This is particularly true for tipping points as our assessment in shows that many of the key variables for the main positive feedback processes are missing entirely in the portfolio.

In the context of tipping points, the prime benefit of remote sensing appears to be in improved system understanding to guide model development. High-resolution (particularly in space, but also in time) monitoring—especially in the ice sheet fringe regions—is essential to inform conceptual and process-based numerical models. From this, parametrisations for whole-ice sheet models can be derived (Benn et al. 2017), providing insights into larger parts of the system. In terms of early warning, the time scales of ice sheet dynamics likely prohibit meaningful predictive power of critical slowing down indicators in the near future. However, transient forecasts and the lessons learned from the recent accelerated ice sheet retreat history allow for improved estimates of the short-term future of components of the ice sheets, such as Pine and Thwaites glaciers. To this end, the concept of propagating tipping (see introduction) is becoming increasingly central to the assessment of ice sheet instability: Given that frontal ablation effects are currently major drivers for ice sheet retreat, regional self-amplified melt through MISI or MICI can have far reaching effects propagating much further inland into the glacier (Reese et al., 2018). This lends even more importance to new/updated ECVs that capture the dynamics of the ice sheets at their fringes.

### Amazon Forest System

#### Tipping System and Feedback Mechanisms

The Amazon forest is a system of high regional and global significance. It hosts a great share of global biodiversity, contributes to net global cooling via evapotranspiration and carbon storage, and plays a key role in moisture distribution in the region (Nobre et al., 2021; Flores et al., 2024). A multitude of drivers, most notably land use and anthropogenic climate change, continuously degrade the forest system (Bullock et al. 2020; Lapola et al. 2023).

Amazon rainforest dieback into a degraded or savannah-like state has been identified as a global core tipping element (Armstrong McKay et al. 2022; Lenton et al. 2023; Wang et al. 2023). Central to this dieback is the role of forests in sustaining atmospheric moisture. Remarkably, about 20% of Amazonian precipitation stems from tree transpiration, which is about two thirds of all moisture that is recycled in the Amazon (Staal et al. 2018). The ability to achieve such water recycling stems from a series of feedback mechanisms delicately co-determining the forest extent itself. Forests, particularly through their deep roots, are crucial for maintaining high moisture fluxes to the atmosphere during dry periods (Sakschewski et al. 2021; Singh et al. 2021), which in turn influences local and downstream precipitation within the Amazon (Zemp et al. 2017; Staal et al. 2018). Loss of forest coverage, e.g. through human deforestation, usually reduces evapotranspiration, thus diminishing both local and downwind precipitation. This reduction is compounded by a decrease in tree-produced volatile organic compounds (VOCs), which serve as cloud condensation nuclei, further reducing precipitation (Ehn et al. 2014; Yáñez-Serrano et al., 2021; Sanaei et al. 2023). Additionally, fire aerosol emissions from burning forests also have an effect on rain patterns, potentially inhibiting rainfall and exacerbating drought conditions. Furthermore, the forest's decreased roughness length leads to increased wind speeds, shortening the residence time of moisture in the forest ecosystem and impacting cloud formation (Barkhordarian et al. 2019; Zhou et al. 2021). These factors collectively contribute to higher temperatures and increased drought stress, exacerbated by less evaporative cooling and reduced shading from the forest canopy (Sampaio et al. 2007). This set of feedback mechanisms, collectively acting as a regional vegetation-atmosphere feedback, can start to dry out the ecosystems and help fires to take effect.

The vegetation-fire feedback loop then is a major contributor for locking the respective former forest area into a degraded savanna or low-tree-cover state (Hoffmann et al. 2012; Flores and Holmgren 2021a, b; Drüke et al. 2023). Grass-fueled fires, thriving in open landscapes, help maintain a grass-dominated, often degraded ecosystem. This dynamic not only increases fire probabilities due to a more open canopy and drier understory, but also contributes to greater erosion due to less vegetative coverage and higher wind speeds, further diminishing the remaining forest resilience and possibility of recovery (Spracklen and Garcia-Carreras 2015). Conversely, a closed tree canopy suppresses grass growth by outshading and creating a humid microclimate (Flores et al., 2024).

**Fig. 3:**
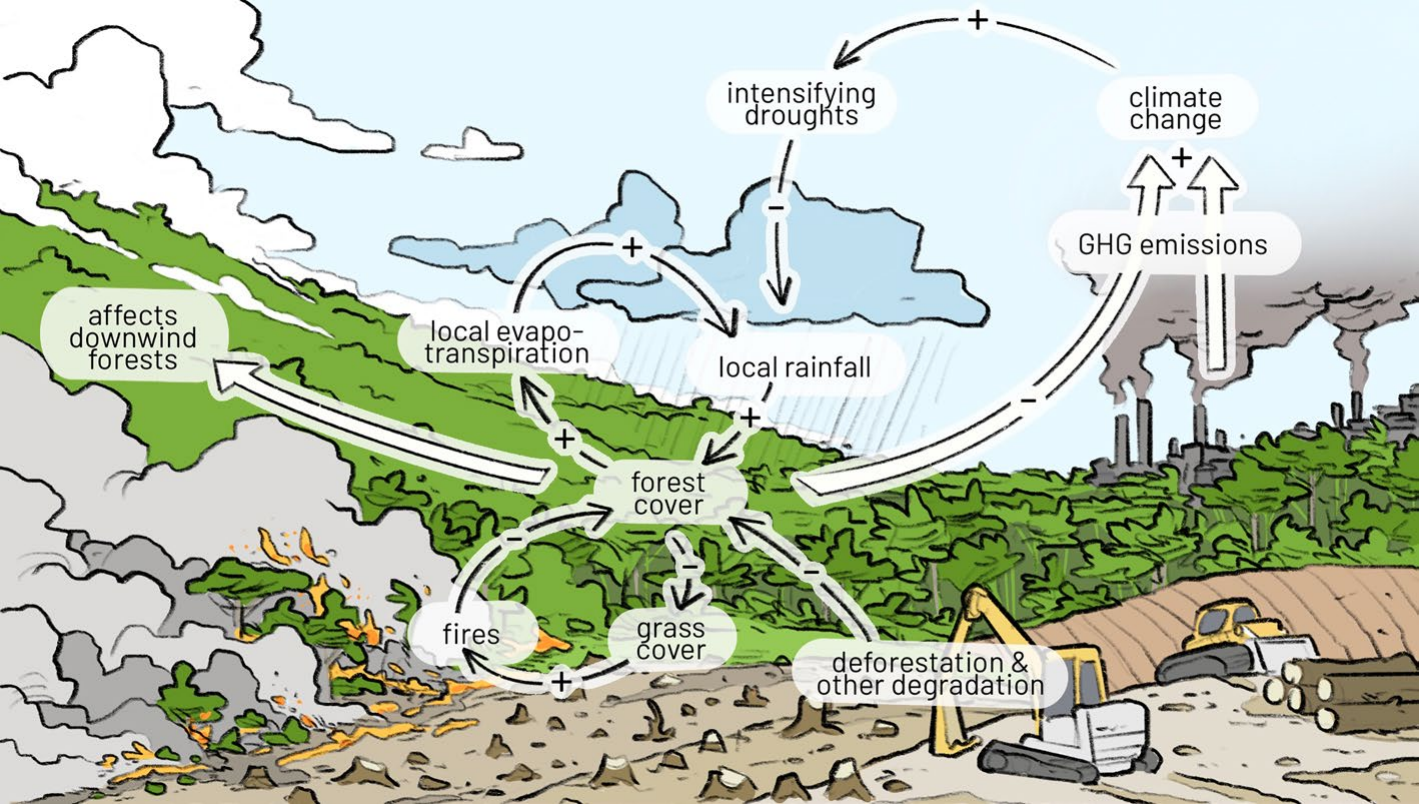
Key feedbacks in the Amazon rainforest are vegetation-rainfall and vegetation-fire

Finally, any form of forest loss, whether through deforestation or degradation, results in significant CO_2_ emissions, whose contributions to climate change are ongoing if the forest is unable to recover and act as a future carbon sink. This cascading effect of forest loss, increased CO_2_ emissions, and exacerbated climate change forms a vicious cycle, perpetuating further forest degradation and loss, thereby disrupting the Amazon's critical ecological balance (Fig. 3).

#### Earth Observation of Tipping

##### Introduction: Monitoring the Amazon Rainforest from Space

Remote sensing of Amazon tipping dynamics requires capturing variables associated with the feedback mechanisms described above. Due to their long operation times Landsat, SPOT-Vegetation, MODIS (and Sentinel-3, Sentinel-2 and Sentinel-1 in the last decade) data are commonly used to study forest cover over time. Landsat time series show that about 17% has been degraded between 1995–2017 as a result of direct human activities and of droughts (Bullock et al. 2020; Lapola et al. 2023).

Beyond forest cover, vertical profiling measurements of carbon compounds using aircraft were carried out over nine years to assess the changing carbon balance of the Amazon and, complemented by remote sensing data, its causes (Gatti et al. 2021). Remote sensing data played a crucial role in providing spatially extensive and temporally consistent information. The combination of data sources enabled a more nuanced and complete understanding of the factors contributing to the Amazon's shift from a carbon sink to a carbon source. For most areas, deforestation was found to be the primary cause. In contrast, in the southeast of the basin, climate change and especially drought was identified as the most important driver. In both cases anthropogenic fires—which are not naturally occuring in tropical forest—can strongly accelerate biomass loss (Basso et al. 2023). Signatures of the vegetation-fire feedback can be detected from space, for instance in MODIS data, where fire occurrence is strongly anti-correlated to forest cover (Staver et al. 2011) and even rises steeply above a threshold around 50% grass cover (Van Nes et al. 2018). Today, observations using MODIS VIIRS, Sentinel-3 and geostationary satellites enable improvements to near real time fire radiative power and smoke detection. But observing understory fire remains a challenge, and the resolution of sensors (Copernicus Atmosphere Monitoring Service (CAMS) products from the Global Fire Assimilation System (GFAS) available are about 11 km resolution globally) constrains early detection of small fires (Andela et al,. 2022).

Building on the findings of Amazon carbon sink loss, Staal et al. (2023) showed with a moisture tracking model that about half of all the land-derived precipitation in the net carbon source area in the Amazon is derived from moisture that evaporated from this same region. This implies a positive feedback in which reduced photosynthesis due to drying leads to further drying in the same precipitation-limited portion of the Amazon. Moisture tracking models reconstruct moisture fluxes from evaporation source to precipitation sink (e.g. Van der Ent et al. 2014; Tuinenburg and Staal 2020) hence aim at quantifying the vegetation-atmosphere feedback. More specifically, these models are forced with three-dimensional wind fluxes, atmospheric moisture content and evaporation and precipitation fluxes from ERA5 (Tuinenburg and Staal 2020) and therefore indirectly integrate remote sensing data. Recently, differently complex models were able to show effects of deforestation on precipitation reduction (Smith et al. 2023, Araújo et al., 2023).

In general the integration of diverse techniques of remote sensing and modelling are becoming more important to study the status and potential tipping of the Amazon. Sentinel-2's capacity to map plant functional diversity (Ma et al. 2020) and GEDI's lidar-based models for biomass density (Duncanson et al. 2022,) provide essential insights into forest health indicators, putting emphasis on biodiversity-ecosystem functioning relationships in the Amazon (Poorter et al. 2015; Sakschewski et al. 2016). Further, the synergy of remote sensing with dynamic forest models (Hiltner et al. 2022), and novel methodologies like radar-based forest productivity estimation (Henniger et al 2023) and lidar-derived tree size distribution (Taubert et al. 2021), enrich our understanding of forest dynamics. The significance of border effects in biomass estimation (Knapp et al. 2021) and the role of forest structure in biomass and productivity (Exbrayat et al. 2019) highlight the importance of diverse and contextual measurements, and data precision. Additionally, remotely sensed functional diversity (Pacheco-Labrador et al. 2022) enables study of forest composition and resilience over space and time. Moreover, integrating multi- and hyperspectral data from numerous in use satellites such as PRISMA, EnMAP, Sentinel-2 and upcoming missions such as NASA’s Surface Biology Geology mission, to be harmonised with ESA’s new CHIME mission, will provide detailed spectral information enhancing our understanding of all points above and give a new level of detail for the physiological status and stress responses of tropical forest (Qian 2021). Taking a holistic approach, leveraging lidar, radar, and multispectral data, offers a comprehensive framework for predicting and managing forest tipping points, informed by a multi-dimensional perspective on ecosystem health. Remote sensing in combination with different modelling techniques offers the opportunity for near real-time tipping point observation systems for the Amazon basin and beyond (Table 2).

**Table 2:** Parameters relevant to Amazon rainforest tipping, and corresponding GCOS-ECVs

Variable related to tipping dynamics	GCOS-ECV parameters		Comment/gap(s)	Suitable?
	Variables	Specs		
**General forest state**				
Forest Cover	Land Cover High resolution land cover IPCC land classes	Threshold 5 years at 1 km, goal monthly at 100–300 m Threshold 5 years at 30–100 m, goal monthly at < 10 m Threshold 5 years at 1–100 km, goal monthly at 10–300 m	Weekly required Land use change	X
Land cover fractions*				
Soil moisture*	Surface soil moisture Root zone soil moisture	Threshold 2 days at 50 km, goal 6 h at 1 km	Surface soil moisture only from satellite records	X
Vegetation structure	–	–	Tree height (maybe height classes and understory distinction), Tree Cover, Tree density are not specified by GCOS but are noted: additional canopy structure variables would be helpful	M
Vegetation composition	–	–	Functional diversity (measureable tree traits), species composition	M
Vegetation water content	–	–	not included	M
Photosynthesis*	Leaf area index (LAI), Fraction of absorbed photosynthetically active radiation (FAPAR)	Threshold 10 days at 250 m, goal daily at 10 m		
Biomass*	Above ground biomass (AGB)	Threshold 5 years with 1 km, goal bi-annual and 10 m	Weekly required Tree height, Tree Cover, Tree density are not specified by GCOS but are noted: additional canopy structure variables would be helpful	X
GHG emissions (CO2 dynamics)*	CO2 mole fraction CO2 column average dry air mixing ratio	Threshold bi-annual and 2000 km, breakthrough monthly, goal daily and 100 km Threshold biweekly and 10 km, goal daily and 1 km	Daily required	X
Albedo	Albedo	Threshold 5 days at 250 m, goal daily at 10 m		
**Particularly for vegetation-atmosphere feedback**				
Evapotranspiration	Evaporation from land (includes Sensible heat flux, Latent heat flux, Bare soil evaporation, Interception loss, Transpiration)	Threshold daily and 25 km, goal hourly and 100 m	Relates to monitoring of precipitation, newly included into 2022 ECVs	
Precipitation	Precipitation in either liquid or solid form	Threshold daily and 25 km, goal hourly and 100 m; Threshold uncertainty 5 mm, goal 1 mm	Accurate measurements are needed (uncertainties need to be met)-related metrics including dry season length and maximum climatological water deficit are key variables for monitoring Amazon tipping	X
**Particularly for vegetation-fire feedback**				
Fires (fire activity, Burned Area)*	Burned area Active fire Fire radiative power (FRP)	Threshold monthly at 1 km, goal daily at 10 m Threshold twice daily at 5 km, goal 5 min at 50 m		

A star (*) indicates what is directly addressed in ESA CCI baseline projects. The last column indicates where the GCOS-ECV parameters do not match the specific needs of tipping science either by insufficient or undefined specs (X) or entirely missing ECV (M)

##### Monitoring the Amazon Rainforest’s Loss in Resilience

Loss in resilience, quantified by appropriate metrics (Bathiany et al., 2024), can serve as an early warning indicator for approaching a tipping point (Dakos et al. 2024). In general, global-scale remote sensing is useful for characterising changes in disturbance regime e.g. for fire, from breakpoints in time-series (Finer and Mamani, 2022), and response via forest resilience assessment from time series of vegetation products and indices (Bathiany et al., 2024). However, remotely sensed burned area is highly spatial resolution-dependent. Of the EO products currently available, burned area, relativized burned area (for fire severity), fire radiative power, land cover, land surface temperature, precipitation, and soil moisture, could be used to distinguish old growth forests that store the most carbon. Above-ground biomass can be a proxy for vegetation structure and water content, and red-edge information from hyperspectral imaging can indicate vegetation health. It is possible to distinguish types of deforestation and attribute the causes by combining optical and SAR (Sentinel-2 and Sentinel-1) imagery: recent innovations based on deep learning classification techniques show promising results in terms of accuracy and usefulness (Slagter et al 2023).

For process understanding, multispectral EO can be used to track the impacts from compounding disturbances and feedbacks at play, i.e. the combination of impacts from droughts, wildfires, deforestation and other human-driven degradation (see for instance Lapola et al. 2023). Compounding disturbance effects are one of the major research challenges, addressing their synergies, to inform efforts to counter their impacts.

The resilience indicators developed so far for the Amazon rainforest are still too uncertain to be useful as EWS, with different conclusions drawn depending on the data product and respective bands and sensors used. For instance, based on K-band Vegetation Optical Depth (VOD), 75% of the Amazon rainforest were categorised as losing resilience since 2003, meaning that such forests could be approaching a local-scale tipping point (Boulton and Boers, 2022). However, VOD may be contaminated by soil moisture: analogous analyses using QSCAT and ASCAT active radar products (which are less sensitive to soil moisture and flooding, and able to penetrate the canopy for vegetation moisture change) have not found signals of resilience loss (Tao et al. 2023), although still actively debated (Boulton and Smith, 2023).

Complementary avenues towards monitoring the Amazon’s resilience comprise tracking species diversity, in particular functional diversity (Sakschewski et al. 2016). Indicators related to functional diversity and richness show great promise for tipping EWS at local to regional-scales, but development of these products, for example from high-resolution hyperspectral imagery or active lidar, relies on the availability of large amounts of accurate in situ data to improve the data quality and uncertainty assessment, so is not currently feasible at large-scale (Jetz et al. 2016; Schneider et al. 2020).

##### Discussion: Gaps in GCOS and Recommendations

The review above shows that a wide range of parameters need to be observed across the Amazon to capture the complexity of the potential tipping system. Relevant variables include burnt area, leaf area index (LAI), vegetation optical depth (VOD), albedo and surface roughness, normalised difference vegetation index (NDVI), cloud cover, LST for plant stress, vegetation water content, soil moisture, and canopy structure indicators such as tree height, tree cover and tree density. For carbon storage in forests biomass is needed: GCOS includes this but the temporal specifications do not yet meet requirements for this specific application. Tree height, tree cover and tree density are not considered by GCOS. Land cover maps need to become more dynamic (regular updates e.g. weekly) via cover fractions such as tree cover, while indicating the drivers of change (such as land use change drivers). Vegetation water content is indicative for forest state but is currently also not considered by GCOS. There is also an urgent need for more comprehensive measurement of stocks, fluxes and atmospheric CO_2_ in humid tropical forests (Friedlingstein et al. 2022; Crisp et al. 2022) as there are large uncertainties in the carbon fluxes between atmosphere and land/ocean reservoirs.

In total, the complexity of the system and the possibility to leverage remote sensing for all stages of the tipping process necessitate the observation of a wide range of variables at high resolution. We contrast the observational requirements from the perspective of tipping points science with the specifications from GCOS and find that many key variables are either missing or insufficiently resolved.

### Atlantic Ocean Circulations

#### Tipping System and Feedback Mechanisms

Two major ocean circulations in the North Atlantic are considered as potential tipping systems (Armstrong McKay et al. 2022; Lenton et al., 2023). The Atlantic Meridional Overturning Circulation (AMOC) transports warm water in an upper surface branch from the equator to the North Atlantic, where it cools, sinks, and returns southwards in a lower deepwater branch. Thereby, the circulation is playing a key role in marine ecosystems and climate, connecting remote regions of the Earth. A slowdown or collapse would reduce the northward heat transport and cause a cooling of the North Atlantic and Europe, as well as a weakening of the monsoon systems and would result in sea level rise along the eastern coast of North America. The subpolar gyre (SPG) circulation south of Greenland constitutes another potential tipping system linked to potential collapse of convection in the Labrador and Irminger seas (Sgubin et al. 2017; Swingedouw et al. 2021). Such a rapid change in overturning would entail significant regional cooling, impact European weather and shift circulations such as the jet stream and the intertropical convergence zone. The critical global warming threshold for tipping has been estimated at about 1.8 °C (with a range 1.1 to 3.8 °C), with a decadal collapse timescale (Armstrong McKay et al. 2022).

**Fig. 4:**
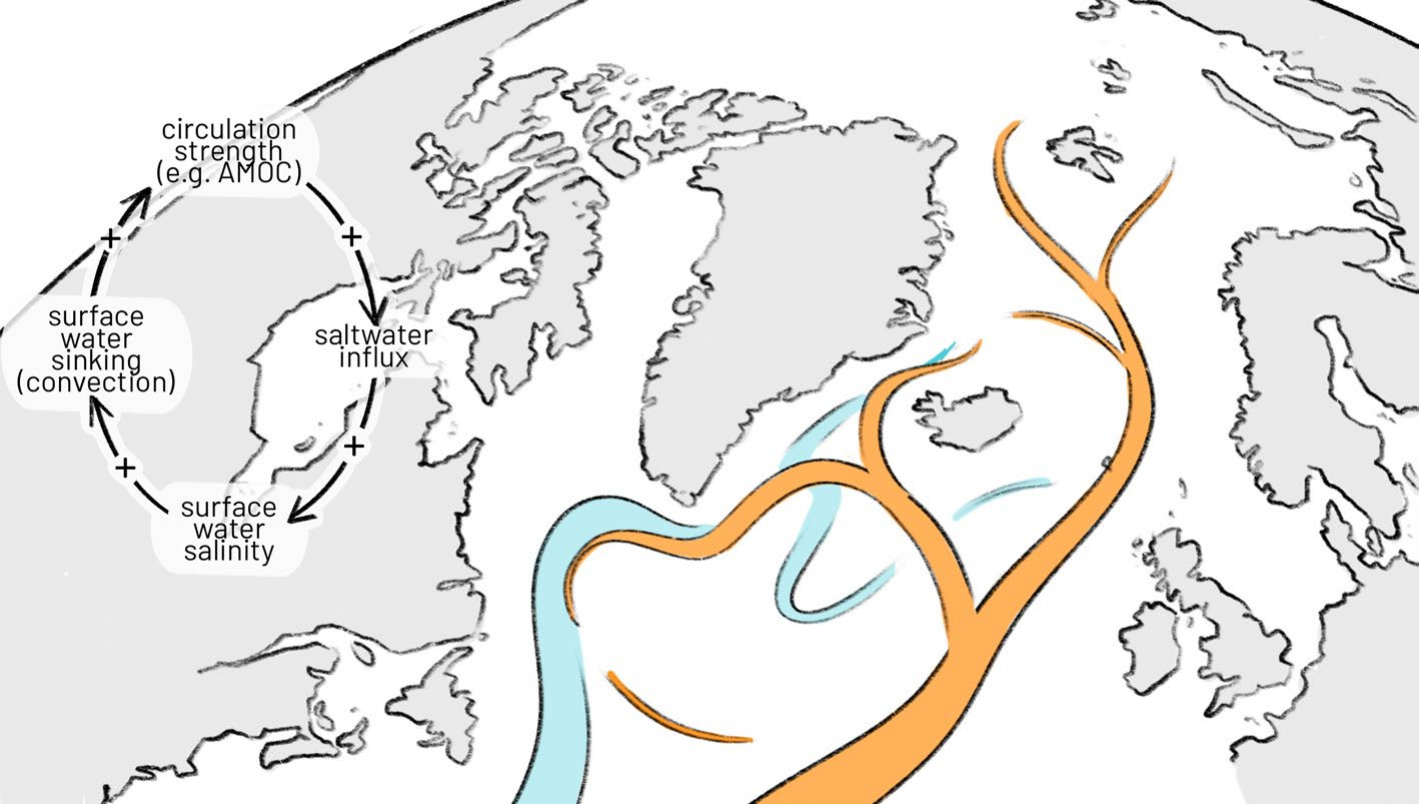
The salt-advection feedback as one key positive feedback driving the Atlantic Meridional Overturning circulation and Subpolar Gyre, based on Loriani et al., (2023)

Both systems are self-sustained due to a salt-advection feedback—in the “on”-mode of the circulations, the import of salty water sustains a sufficient surface salinity for convection, thereby continuing to drive the overturning circulation. This process is however susceptible to freshwater influxes, e.g. through increased ice sheet melt, precipitation or river influxes. The induced decrease in surface water salinity reduces the sub-polar water column density and weakens the convection, thereby the overturning strength and saltwater import, again decreasing the surface water salinity. Other, potentially negative feedbacks are related to interaction with sea ice, transport of freshwater, interactions with the atmosphere have in parts stabilising effects (Jackson et al., 2017; Wood et al. 2024) (Table 3) (Fig. 4).

#### Earth Observation of Tipping

##### Introduction: Monitoring the Ocean Circulations from Space

Wood et al. (2024) provide an in-depth overview on the opportunities and merits of Earth observation for monitoring the AMOC and SPG. In short, our best tool for understanding potential tipping in the overturning circulations are climate models of varying complexity, ranging from early box models to high-complexity coupled climate models (Stommel, 1961, Jackson et al. 2023). Direct observations of overturning strength are so far limited to in situ hydrographic arrays providing continuous measurements since 2004 in selected locations (Swingedouw et al. 2020).

However, a range of remote sensing approaches can support the monitoring (Wood et al. 2024; Swingedouw et al. 2020). Leveraging decades of satellite observations with wide spatial coverage, the hydrographic time series have been extended in time and space via altimetry of sea level (Frajka-Williams, 2015; Mercier et al. 2015) and via gravimetry of density variations in the deep ocean (Landerer et al. 2015). Complementary approaches assess proxies of the overturning strength, e.g. by determining the dynamics at the convection sites by inferring surface density from measurements of sea surface temperature (SST) and sea surface salinity (SSS; Swingedouw et al. 2020). SST and SSS are monitored by radiometers at microwave (e.g. on GMI, SLSTR, AVHRSS) and L-band frequencies (sensitive to salinity-induced changes in the di-electric constant of seawater; e.g. on SMOS or planned CIMR), respectively. The observed changes in spatial and seasonal SST (the ‘warming hole’ south of Greenland) have been attributed to an AMOC slowdown (Caesar et al. 2018) or SPG weakening (Sgubin et al. 2017; Swingedouw et al. 2021), although remote influences may also play a role in creating such SST patterns (e.g. Hu and Fedorov 2020).

The time span covered by hydrographic arrays or even satellite observations is, however, too short to exploit statistical properties such as temporal autocorrelation as early warning signatures (stage 2; see Lobelle et al. 2020; Boulton et al., 2014). Therefore, several studies rely on long-time paleo proxies or historical instrumental observations for temperature and salinity to infer AMOC weakening (Moffa-Sánchez et al. 2019; Caesar et al. 2021) or approaching criticality (Boers 2021; Ditlevsen and Ditlevsen 2023; Michel et al. 2022). There are however ongoing debates about potential false warnings (Michel et al. 2023) and the feasibility of these proxies for AMOC tipping in general (Kilbourne et al. 2022; Jackson and Wood 2020). One main difficulty is that the assessment of potential bistability of both AMOC and SPG and potential proximity to a critical threshold depends on good representation of the non-linear feedbacks in the models (Jackson et al. 2023). This is potentially constrained by biases in stratification (Swingedouw et al. 2021), salinity (Liu et al. 2017; Mecking et al. 2022) or limited resolution of eddies (Heuze, 2017; Swingedouw et al., 2021) and lies at the focus of current and upcoming modelling initiatives like NAHosMIP (Jackson et al. 2023) and TIPMIP.

Concerning the salt-advection feedback, the influx of fresh water plays a key role. Therefore, a significant leverage of remote sensing data for the Atlantic ocean circulations lies in the monitoring of the mass balance of the Greenland Ice sheet (IMBIE 2020, Dukhovskoy et al. 2021), discharges from Arctic rivers (Druckenmiller et al., 2021) and connected basins such as the Beaufort Gyre (Zhang and Thomas 2021; Lin et al. 2023) as well as atmospheric circulation changes (Holliday et al. 2020) and sea ice (Drijfhout et al. 2015; Sgubin et al. 2017), and through combination with hydrographic and fixed array data, oceanic fresh water transports (McDonagh et al. 2015) (Table 3).

**Table 3:** Parameters relevant to AMOC/SPG tipping, and corresponding GCOS-ECVs

Variable related to tipping dynamics	GCOS-ECV parameters		Comment/gap(s)	Suitable?
	Variables	Specs		
**Salt-advection feedback and overturning proxies**				
Sea surface temperature*	Sea surface temperature	Threshold 100 km, weekly, goal 5 km, daily	HIgher resolution is needed for eddies at threshold 10 km, goal 1–3 km, at least in key boundary/choke point regions	X
Sea surface salinity*	Sea surface salinity	Threshold 50–100 km and weekly, goal daily at 10 km	Higher resolution valuable in key boundary and choke point regions	X
Sea surface height*	Global mean sea level	Threshold 100 km and monthly (weekly at regional scale), goal 10 km and daily;	Detection changes in overturning; Regions for higher sampling undefined	?
Ocean section freshwater transports	–	–	Derived mainly from fixed arrays/Argo. EO or reanalysis data for wind stress needed to obtain transport estimates	M
**Other influence on overturning strength**				
Sea ice concentration*	Sea ice concentration	Threshold 50 km and monthly, goal 1 km and better than daily	High resolution particularly in key boundary and choke point regions	X
Sea ice thickness*	Sea ice thickness	Threshold 50 km and monthly, goal 1 km and daily	High resolution particularly in key boundary and choke point regions	X
Sea ice motion	Sea ice drift	Threshold 50 km and monthly, goal 1 km and daily	High resolution particularly in key boundary and choke point regions	X
Wind change	Surface currents			
Precipitation over the ocean	Accumulated precipitation	Threshold 250 km, Goal 50 km. Threshold time scale yearly, Breakthrough Monthly	Monthly time resolution allows resolution of seasonal convection in subpolar gyre. Need consistency with evaporation—precipitation is the more challenging of the two	X
Evaporation over the ocean	Latent heat flux	Threshold 100 km, Goal 10 km		
Freshwater input—rivers	River discharge	Annually to daily	Gauging station specific. Consistent basin scale estimates needed to construct fresh water budgets. Accuracy needs to be consistent with precipitation minus evaporation, and basin scale fresh water content	X
Freshwater input—ice edge melting rate	–	–	Not defined	M

A star (*) indicates what is directly addressed in ESA CCI baseline projects. The last column indicates where the GCOS-ECV parameters do not match the specific needs of tipping science either by insufficient or undefined specs (X) or entirely missing ECV (M)

##### Discussion: Gaps in GCOS and recommendations

Despite the outlined challenges and uncertainties about EO use for direct monitoring of AMOC and SPG, it serves as an indispensable component at the interplay of models, in situ measurements and evidence from paleo records. Based on the key variables in the salt-advection feedback and current approaches to reconstructing overturning strength (including proxy-based methods), several direct surface quantities need to be monitored, that is sea surface salinity, temperature and elevation. All of them are included in the GCOS ECVs with largely sufficient specifications with the exception of spatial resolution for eddy-resolving modelling, and coverage needs to be improved at high latitudes (Estella-Perez et al. 2020). Sea surface salinity can be also indirectly estimated through freshwater influx (precipitation—evaporation, North Atlantic river runoff, melting of glaciers and sea ice). This calls for improved resolution analyses near coasts and for estuaries, with special attention to key regions such as the Fram and Davis straits and subpolar basins (Wood et al. 2024). Sea surface height analyses using satellite altimetry and density change through gravimetric observations have provided added value (Swingedouw et al. 2020). The recent discussions around the suitability of a range of proxies show that the main value of EO for AMOC/SPG tipping lies in the wide spatial coverage of multiple variables, offering potential in resolving tipping fingerprints in multivariate analyses (Michel et al. 2023). The GCOS ECVs should continue to be scrutinised with respect to potential additional variables useful for tipping research. GCOS currently considers the majority of so far identified parameters of relevance for AMOC monitoring. Density change and freshwater input are, however, not addressed. A potential area for further development would be to monitor integrated regional ocean heat and fresh water budgets in key regions such as the subpolar gyre. This will need a combination of in situ (Argo, fixed section arrays) and EO data, and a focus on specifying *combinations* of ECVs to achieve consistent accuracy for budget calculation of such integral quantities (e.g. regional heat content and section-integrated ocean transports) (Forster et al. 2021).

### Permafrost

#### Tipping System and Feedback Mechanisms

Permafrost is defined based on ground temperature (Matthews et al. 2021). Values below zero degree Celsius need to occur for at least two consecutive years. This excludes the upper soil (active layer) which thaws and refreezes seasonally. Permafrost is thus a subsurface phenomenon. The discussion in the context of climate tipping elements centres around the release of soil carbon which is currently still frozen (Swingedouw et al. 2020).

If ground thaws, subground carbon becomes available for decomposition and release as CO_2_ or CH_4_, depending on soil saturation, resulting in temperature increase. This leads to enhanced thaw and subsequent carbon release and is expected to translate into a range of 0.13–0.27 °C additional global warming by 2100 (Schuur et al. 2015). Permafrost thaw has only recently been discussed as a tipping system under abrupt thaw conditions. Otherwise greenhouse gases are expected to be gradually released with permafrost thaw with quasi-linear behaviour (Lenton et al. 2008; Schuur et al. 2015; Nitzbon et al. 2024). It has been suggested that microbial properties are also important, as when activated, microbial organisms are expected to cause additional warming and increased thaw (Bathiany et al. 2016; Steffen et al. 2018). This effect is expected to become relevant at a threshold rate of local warming (Luke and Cox, 2010). The hydrological feedback between permafrost and climate has also been suggested to be relevant to tipping science recently (Brovkin et al. in review, this issue). Moisture in the ground and seasonal storage in overlying snow influences heat transfer. The feedback magnitude is less well studied than the biogeochemical feedback, but is essential also considering teleconnections.

Localised positive feedback mechanisms have been identified related to ground thaw (Armstrong McKay et al. 2022). Abrupt thaw which is often triggered by unusually warm summers results in terrain change. This includes ground subsidence (immediate loss of ice in ground) and thaw slumps (permafrost specific landslides) (Brovkin et al. in review, this issue). Localised tipping occurs in clusters synchronised across large areas (Lenton et al. 2024). This has been specifically investigated for permafrost lakes for the last two decades (Nitze et al. 2018).

#### Earth Observation of Tipping

##### Introduction: Monitoring Permafrost from Space

Subground temperature is essential for monitoring permafrost state. Monitoring requires in situ monitoring with boreholes, which are of limited availability and representativeness (Biskaborn et al. 2015). Satellite observations are used to complement the in situ measurements through modelling and land surface proxies (Trofaier et al. 2017; Bartsch et al. 2023a, b). Current approaches rely on models that utilise satellite-derived land surface temperature from thermal sensors which are impacted by cloud cover (e.g. Obu et al. 2019). Records are therefore gap-filled based on reanalysis data. Insulation/heat transfer properties are considered mechanisms in the modelling, specifically properties of snow (snow water equivalent) and soils (Westermann et al. 2015, 2023). Land cover is used as a proxy, specifically to separate areas with peat and wetlands. Common soil moisture detection schemes using microwave satellite data fail due to land cover heterogeneity (Högström and Bartsch 2017; Högström et al. 2018; Wrona et al. 2017).

For actual assessment of tipping with respect to release of soil carbon, GHG emissions need to be quantified. Advanced satellite missions as well as improved retrieval, adjusted to the Arctic, are needed to facilitate monitoring at the required detail (Miner et al. 2022). The added value of remote sensing in this context is currently comparably low (Swingedouw et al. 2020; Lenton et al. 2024). The potential lies largely in identification of localised tipping and abrupt thaw detection.

Land surface characteristics including lake extent change and subsidence are commonly used as indicators for local tipping (abrupt thaw features) and permafrost thaw in general. Regional clusters of ground thaw can be identified through land cover change reliably for up to 20 years back in time (Nitze et al. 2018; Runge et al. 2022). The investigation of abrupt thaw features which results in terrain change (thaw slumps) requires availability of terrain information at high spatial resolution. The ground also subsides where ice is lost in the ground due to thaw: this can be be quantified with synthetic aperture radar (SAR) data if long-term continuous records exist (e.g. Strozzi et al. 2018). However, soil organic carbon (SOC) content needs to be known to assess the potential magnitude of feedbacks. Interpolated maps from in situ records are currently the main source for SOC. They lack detail and consistency across the Arctic (Bartsch et al. 2016a, b). Land cover and land surface properties, as identified from space using multispectral as well as SAR data, have been to date only partially used to fill the gaps (Hugelius et al. 2013; Bartsch et al. 2016a) (Table 4).

**Table 4:** Parameters relevant to permafrost tipping, and corresponding GCOS-ECVs

Variables related to tipping dynamics	GCOS-ECV parameters		Comment/gap(s)	Suitable?
	Variables	Specs		
**Ground thaw—carbon release feedback**				
Ground temperature*	Thermal state of permafrost Active layer thickness	Sufficient sites to characterise each bio-climate zone, in situ specific specifications	Specifications not applicable for satellite based retrievals	X
	Land surface temperature	Threshold 1 km (at 6 h), Goal < 1 km	Goal spatial resolution insufficiently defined	X
	Soil temperature	Down to 180 cm, Threshold 139– 278 km daily, Goal 5 km at 3 h	Vertical and spatial resolution requirements do not address permafrost needs	
Soil organic carbon content	Soil organic carbon content % of organic carbon in the topmost 30 cm and sub-soil 30–100 cm	20 to 1000 km (20 to 1000 m for peatlands + plus mineral soil bulk density)	Peatland specifications applicable Currently satellite-derived products meeting the requirements are only locally available	X
GHG flux*	CO2 mole fraction/column average CH4 mole fraction/column average	100–2000 km for mole fraction 1–10 km for column	Limited in situ availability for permafrost, constraints for satellite retrievals in high latitudes	X
**Misc: linked to permafrost state**				
Thermokarst lake water extent	Lake water extent	Threshold monthly at 1 km, goal 5 days at 10 m	Lake drainage as indicator of thaw, Threshold spatial requirements insufficient, goal requirements applicable	X
Surface elevation change	–	–	Long-term subsidence required as indicator of thaw High spatial detail needed for abrupt thaw feature monitoring	M
Snow water equivalent*	Snow water equivalent	Threshold every 2nd day at 25 km, goal 6 h at 0.5 km	Insulation effect, Threshold spatial requirements insufficient	X
Land cover*	Land cover high resolution land cover	Threshold 5 years at 1 km, goal monthly at 100–300 m Threshold 5 years at 30–100 m, goal monthly at < 10 m	Abrupt thaw indicator Only high resolution land cover applicable	X
Soil moisture	Surface soil moisture Root zone soil moisture	Threshold 2 days at 50 km, goal 6 h at 1 km	Too coarse to represent Arctic landcover heterogeneity; Surface soil moisture only from satellite records, and operational schemes fail at high latitudes	X

A star (*) indicates what is directly addressed in ESA CCI baseline projects. The last column indicates where the GCOS-ECV parameters do not match the specific needs of tipping science either by insufficient or undefined specs (X) or entirely missing ECV (M)

##### Discussion: Gaps in GCOS and Recommendations

Specifications dedicated to permafrost monitoring using satellite data are lacking for GCOS (see Table 4). Only in situ monitoring is addressed. Specifications under the more generic ECV soil temperature are not applicable in the current form. In case of lake extent, land cover and snow water equivalent, goal requirements need to be met to be applicable for permafrost. Ground subsidence is regionally feasible and can be potentially fully implemented on for the entire permafrost domain but is not considered as an ECV for land (only for glaciers).

### Other potential tipping systems

#### Lakes

Although lakes and freshwaters in general are not considered tipping elements in the climate system (Lenton et al. 2008; Armstrong McKay et al. 2022), lake shifts provide important direct or indirect feedbacks to the Earth’s climate system. Among others, abrupt lake water level or extent changes directly affect water availability with direct consequence for vegetation and thus respiration (Richardson et al. 2013). Changes in the capacity of lakes to store carbon in their sediments could affect the carbon budget of lakes and biogeochemical alterations in lakes could lead to changes in the exchange of greenhouse gases between lakes and the atmosphere (Bartosiewicz et al., 2021). More studies should explore the climate–hydrology–biogeochemistry linkages that lead to lake ecosystem shifts and potential feedbacks to the climate system (Lehnherr et al. 2018).

Climate pressure can push lakes towards tipping points with important consequences. One of the typical lake shift analyses is the transition from phytoplankton- to macrophytes- dominated state of lakes (Scheffer 1991, 1993, 2001; Loverde-Oliveira et al. 2009; Kosten et al., 2012). However, more and more studies are nowadays focusing on other lake shifts such as shifts in lake mixing regime (Woolway and Merchant 2019) and the cascading effects on oxygen regime (Schwefel et al 2016). Surface warming increases the stability of the water column of lakes resulting in longer periods of stratification. Longer stratification means reduced mixing and thus less exchange between epilimnion and hypolimnion. As a result, the nutrient as well as the oxygen transport along the water column are reduced with consequences for the lake biota as well.

As for other tipping systems, feedback mechanisms can be identified in lakes. A recent review on the physical trends related to increased duration and strength of density stratification, and the related chemical and biological consequences (Mesman et al. 2021) found that the internal feedbacks can reinforce (positive feedbacks) or slow down (negative feedbacks) shifts between mixing regimes in lakes that stratify at least during one season. Lake internal feedback can be different depending on the mixing regime of lakes (and within these classes the morphometry of the lake can also differentiate the feedback strength). Lake internal feedback could stabilise and even determine the mixing regime, especially in situations where morphometry and climate can support multiple mixing regimes. For example recent studies predicted shifts from dimictic to monomictic regime, where ice cover and inverse stratification in winter are disappearing; and from holomictic to oligomictic or meromictic regime in non-ice covered lakes (Shatwell et al. 2019).

The main source of data to analyse lake shifts are of four different types: in situ measurements, paleolimnological records, models or satellite remote sensing (Calamita et al. 2024); all with different spatial and temporal resolution and different timespan. EO data has been mainly used for studies about two types of lake shifts: lake water extent (Buma et al. 2018; Liang and Li 2019; Nitze et al. 2018; Bai et al. 2021; Zhang et al. 2021), and ice cover (Sun et al. 2018; Wang et al. 2018; Liu et al., 2020). Both are considered ECVs by GCOS and requirements are specified. They differ between the two parameters regarding goal specifications for horizontal resolution, 10 m versus 50 m, but threshold is 1 km for both. However, the scope of usage of EO in the field of lake shifts has broadened in recent years (Calamita et al. 2024). EO has been recently used also to detect and analyse ecological shifts (Bergamino et al. 2007; Duan et al. 2014; Free et al. 2021) and shifts in mixing regime (Woolway and Merchant 2017, 2018; Fichot et al. 2019). This includes observations of chlorophyll-a concentrations (Bergamino et al. 2007; Free et al. 2021). The ECV ‘lake water-leaving reflectance’ is expected to support retrieval. An additional parameter is surface concentration of particulate organic carbon (POC; Duan et al. 2014) which is currently not addressed through GCOS. Monitoring of mixing shifts with remote sensing is addressed through analyses of lake surface water temperature. Goal and threshold requirements for temporal and spatial scale have been defined for this ECV but are not well justified.

#### Ocean Deoxygenation

The dissolved oxygen ocean content has decreased over the last 50 years by about 2% (Schmidtko et al. 2017), reflecting climate-driven changes in ocean circulation and ventilation and changes in biological oxygen demand. Ocean deoxygenation is a severe threat to life in the ocean, impairing vital functions, causing mass mortality and habitat compression (Breitburg et al. 2018) and affecting the microbially-mediated cycling of key elements, mostly carbon (C), nitrogen (N), phosphorus (P) and iron (Fe). Under low oxygen conditions the process of denitrification can lead to a net loss of fixed nitrogen and reduced ocean fertility, with potential impact on ocean productivity and biologically-driven carbon storage. Further, ocean deoxygenation affects the oceanic production of a potent greenhouse gas N_2_O which is strongly enhanced under low-oxygen conditions, potentially providing a positive feedback to global warming.

Ocean deoxygenation is currently regarded as an uncertain potential tipping system (Armstrong McKay et al. 2022), due to the large uncertainties over drivers, mechanisms, time-scale and thresholds for triggering a widespread oxygen loss in the ocean (for more detail see Wood et al. 2024). Given the profound implications that oxygen changes have on marine ecosystems and their services, O_2_ is identified as an ECV by GCOS (WMO, 2022a), and it is of high priority to reliably monitor and assess the likelihood of widespread ocean deoxygenation under current global warming.

Observations from multiple observing platforms (ship-based, fixed-point moorings, autonomous like BGC-Argo, gliders, AUV) key for identifying ongoing subsurface ocean deoxygenation trends and for assessing intraseasonal, seasonal and interannual oxygen variability. Although the oxygen-observing network is expanding (Grégoire et al. 2021), and machine learning (ML) is increasingly used as a powerful method to fill the observational gap (eg. Sharp et al. 2023), further improved observational coverage, spatial and temporal resolution is needed to meet the GCOS requirements to better monitor and quantify oxygen changes (WMO, 2022a). Satellite products are becoming increasingly used for assessing climate change as well as for the near-real time monitoring of ocean health (e.g. Groom et al. 2019; Vinogradova et al. 2019). Yet, their use in support of monitoring ocean deoxygenation and identifying areas susceptible to hypoxia is still limited. This is due to the lack of a direct EO-observed proxy for ocean oxygen and limited vertical coverage of EO to the surface ocean. However, machine learning (ML) algorithms have been developed to reconstruct 3D field of physical (e.g. temperature and salinity; Buongiorno Nardelli 2020) and biological variables (e.g. chlorophyll; Sammartino et al. 2020) from 2D remote sensing imageries. ML approaches have also been used to reconstruct biogeochemical variables at global scale from Biogeochemical-Argo (BGC-Argo) data combined with satellite measurements (Sauzède et al. 2015, 2016, 2017). Hence, the use of artificial intelligence (AI) in conjunction with Earth observed variables (associated with O_2_ variability) and O_2_ observations appears as a promising avenue towards the reconstruction of O_2_ fields over the satellite era, for monitoring short time scale deoxygenation events (eutrophication) and climate trends. Chlorophyll-a, recognized by GCOS as an ECV, is a proxy of phytoplankton biomass and is used for assessing biological productivity space–time dynamics. The increase in the number of spectral bands, from 4 for Coastal Zone Colour Scanner to 21 for Sentinel 3, has improved the quality of OC products with a better differentiation between chlorophyll-a (Chl-a) and non-organic materials, yet its GCOS requirements are insufficiently defined. Estimation of sea surface salinity (SSS) from space in combination with sea surface temperature (SST) and sea surface height (SSH), offer the possibility to follow the evolution of the sea surface density which can be related to the intensity of the ventilation process, circulation, plume tracking, oxygen saturation level and, in combination with in situ data, stratification dynamics. Although these parameters are included in the GCOS portfolio, their requirements may need to be revised for their suitability to support early warning for ocean deoxygenation. Further potential may derive from indirect proxies of ocean deoxygenation such the oceanic emissions of N_2_O. This GHG has proven difficult to estimate by satellite because of its weak spectral signature in the infra-red band and its low variability in the troposphere (ppb), although retrieval of tropospheric N_2_O concentration profiles may be possible from high spectral resolution nadir measurements (Thermal Infrared 1200–1300 cm-1; AIRS and IASI sensors). However, the low spatial resolution, contamination with other gases (H_2_O, CH_4_), and low sensitivity to tropospheric concentration currently limit the exploitation of this satellite-derived N_2_O product (Garcia et al., 2018). Next generation instruments (IASI-NG) could improve the observation capabilities in this direction (Ricaud et al. 2021).

## Summary of Gaps in GCOS Portfolio and Outlook

### Do the GCOS ECVs Meet the Needs of Tipping Points Science?

A wide range of parameters which are of relevance for tipping processes in the Earth’s climate system are considered for monitoring in the GCOS specifications. The utility of remote sensing is often limited for direct observation of ongoing tipping dynamics or early warning signals of approaching tipping points (Lenton et al. 2024). The main merit rather lies in provision of means for improvement of numerical models of climate tipping systems (with or without data assimilation), thereby allowing for improved understanding of the processes and system state (stage 1). Spatial coverage through satellite data is in most cases an asset. Stage 2 (EWS) observation is mostly constrained because of the relatively short record length of the satellite record (about five decades). Integrated use of multiple sensors and platforms (satellites, aircrafts, drones, ground stations etc.) is therefore crucial in conjunction with modelling and paleoclimate evidence.

The majority of variables needed to monitor key tipping systems are considered as GCOS ECVs. Gaps for key elements have been identified for the Amazon rainforest (vegetation water content), permafrost (ground subsidence), AMOC (section mass, heat and fresh water transports and freshwater input from ice sheet edges) and specifically ice sheets. In the latter case, recognition as an ECV is missing for vertical land motion for isostatic effects, melt area, mass flux across the grounding line, calving rate and areas of hydrofracturing. Vertical motion and melt area are required for monitoring the melt-elevation feedback. Glacier melt area has been also previously reported as required but missing in the context of mountain ECVs (Thornton et al. 2021). In addition, lake properties such as particulate organic carbon and chlorophyll-a are lacking. Most parameters not considered under GCOS for the tipping systems discussed here are also not addressed in the framework of the ESA Climate Change Initiative (CCI) program, showcasing the important implications of whether or not variables are recognised as ECVs. Further on, all considered systems require monitoring of parameters which are (in parts) listed in GCOS but so far not addressed by the CCI program.

For many of the ECVs, issues in specifications (from threshold to goal) have been identified. Of main concern is spatial resolution, but also the means of measurements. This applies specifically for cases where the ECVs refer to subsurface properties which cannot be directly measured from space, but through use in models. For example, GCOS ground temperature specifications for permafrost target only in situ point measurements while the majority of other ECVs, including e.g. soil organic carbon content and sea ice thickness, considers spatially continuous observations through satellite observations. In many cases, the threshold spatial resolution target is insufficient to address tipping, only meeting the goal requirements would ensure applicability. Consistency of specification across several related variables is also an important issue (e.g. to define regional ocean heat/water budgets). Several ECVs are related to lake shifts, including lake water extent and lake ice cover, chlorophyll-a concentrations and lake water-leaving reflectance and lake surface water temperature (LSWT). Goal and threshold requirements for temporal and spatial scale have been defined for LSWT but are not well justified. Surface concentration of particulate organic carbon is missing.

Another issue concerns ECVs that are recognised by GCOS but where the requirements are not well justified for remote sensing or defined (or only in parts). This includes lake surface temperature, permafrost ground temperature, lake and ocean chlorophyll-a concentration, surface elevation of ice sheets and grounding line location. Goal requirements for land cover and biomass do not meet the requirements of Amazon rain forest monitoring.

Monitoring of proxies for actual ECVs is of high relevance in many cases, especially when subground phenomena are investigated. For example in case of permafrost abrupt thaw relevant parameters (thaw lake change, terrain change/ground subsidence) and soil properties are needed to assess the vulnerability of tipping (presence of carbon in the ground). The potential for reaching full spatial coverage with satellite data is limited in these cases since comparably high spatial and temporal resolution is needed. A major constraint for early warning regarding permafrost is the limited availability of CO_2_ and CH_4_ observation in high latitudes. Similarly, the state of AMOC and SPG is challenging to assess from space directly, and reconstructions rely on combinations of models and proxy data such as sea surface temperature and salinity to infer the buoyancy conditions that determine the overturning. Remote sensing shines here by offering a wide range of variables and broad spatial coverage, the utility of which is however determined by the capacity of the models to capture the key tipping dynamics.

Regarding ocean deoxygenation, O_2_ is identified by GCOS as ECV, with various different approaches underway to improve observational coverage to meet the requirements (through AI reconstruction, indirect proxies like N_2_O and SSS, SST, SSH). Revisioning of the requirements of the proxies is needed to support EWS of ocean deoxygenation.

Several parameters are identified as of relevance across several of the discussed elements. The different requirements are not reflected in the GCOS portfolio. For example lake water extent is of relevance for shift in lakes as well as proxy for permafrost (thaw lakes). The spatial resolution needs to be higher than currently defined as goal in both cases. Similarly, monitoring the ice loss at the fringes of the Greenland ice sheet does not only capture the ice sheet state but is also of high importance for stability assessments of the AMOC and SPG.

### Recommendations

In this analysis, we have made first steps towards specifying actual observation needs from a tipping points perspective for future ECV considerations. The discussed tipping systems only present a selection and, looking forward, merit more in-depth studies from the individual domain communities. In light of the very active tipping points research, requirements specifications in the GCOS portfolio need to be continuously revised with respect to emerging tipping systems. However, in an update of essential climate variables it is critical to consider a balance between parsimony and specificity, taking into account the resources required to meet the needs. While our work set out to identify shortcomings in the GCOS-ECV portfolio from the perspective of tipping points science, our findings don’t necessarily warrant closing all these gaps in this portfolio. Rather, subsequent work should identify which of the observational requirements should find their way into the ECVs, and analyse the benefit of a complementary set of essential tipping points variables. In line with similar efforts on (climate) variables for ocean (Miloslavich et al. 2018), biodiversity (Pereira et al., 2013) and mountains (Thornton et al. 2021), the tipping points community could define its needs in an independent portfolio centred around the monitoring of critical, positive feedback loops.

Our analysis needs to be extended to further systems and different angles (numerical and conceptual modelling, paleo reconstruction, in situ observation, etc.) considered to provide a full assessment where monitoring of tipping is limited in terms of observational evidence. The rapid advances in computational capabilities (including high-resolution modelling, big data and machine learning approaches) and continued availability of (open access) data hold the potential for ever-increasing utility of EO for tipping research. We encourage the tipping community to revise what their requirements are in terms of monitored variables, spatial and temporal resolution. This may guide mission and even sensor development for better informed risk assessments with respect to emerging climate tipping dynamics under increasing human pressures in the future.
